# Extranodal NK/T Cell Lymphoma with Destruction of the Uvulae: A Case Report

**Published:** 2017-03

**Authors:** Farahzad Jabbari Azad, Zahra Delavarian, Masoud Hatami, Hosein Rahimi, Mohammad Reza Abdolvahed

**Affiliations:** 1 *Allergy Research Center, School of Medicine, Mashhad University of Medical Sciences, Mashhad, Iran.*; 2 *Oral and Maxillofacial Diseases Research Center, Faculty of Dentistry, Mashhad University of Medical Science, Mashhad, Iran. *; 3 *Department of Oral and Maxillofacial Medicine, Faculty of Dentistry, Mashhad University of Medical Science, Mashhad, Iran.*; 4 *Department of Hematology-Oncology, Mashhad University of Medical Science, Mashhad, Iran.*

**Keywords:** Extranodal NK-T-cell lymphoma, Oral ucer, Palate, Uvula

## Abstract

**Introduction:**

Extranodal Natural Killer (NK)/T-cell lymphoma (NKTCL) nasal type is a rare but well-known disease with poor prognosis. NKTCL is more prevalent in Asia and comprises about 7-10% of all non-Hodgkin lymphoma cases in this region. The characteristic clinical pattern of NKTCL is the destruction of the midline structures of the mid-face.

**Case Report:**

The present study examines a case of NKTCL in a 23-year-old man with a destructive ulcer of the palate and uvulae. Based on immunohistochemical results, after three months of delay, the definitive diagnosis was revealed to be Extranodal NK/T cell lymphoma. Following the third cycle of chemotherapy, the patient died due to sepsis and infection.

**Conclusion:**

It is very common to misdiagnose NKTCL with other clinical conditions such as necrotizing stomatitis, deep fungal ulcers, Wegener’s Granulomatosis disease, etc. Delay in diagnosis can worsen the course of the disease and its prognosis.

## Introduction

Natural killer (NK)/T cell lymphoma, nasal type (NKTCL), previously known as lethal midline granuloma, is a rare Non-Hodgkin Lymphoma (NHL) that manifests in the mid-facial structures in a destructive manner (1). Its extreme association with Epstein-Barr virus (EBV) infection has been approved in previous studies (1-6).

Initial signs and symptoms are often limited to the nasal area; including epistaxis, stuffiness, and rhinorrhea. Early oral manifestations are the swelling of the soft palate and posterior hard palate. Over time, this becomes ulcerated which leads to the destruction of the palate and the formation of oronasal fistulae. The signs and symptoms of NKTCL are non-specific, except for a centrifugal tropism to facial structures, making this a diagnostic challenge. Therefore, the clinician must consider this entity in the differential diagnosis of single chronic destructive ulcers of the oral cavity, such as trauma, Necrotizing stomatitis, squamous cell carcinoma (SCC) and deep fungal ulcers (such as Mucormycosis, etc.). The overall prognosis of NKTCL is poor with 5-year survival rates below 30% in spite of chemo-radiotherapy treatments (7). In this paper we report a case of Natural killer (NK)/T cell lymphoma in a 23-year old Iranian man. To our knowledge, it is the second report of this entity from Iran following the report of Moradi et al. which examined palatal ulcers (8).

## Case Report

**Fig 1:**
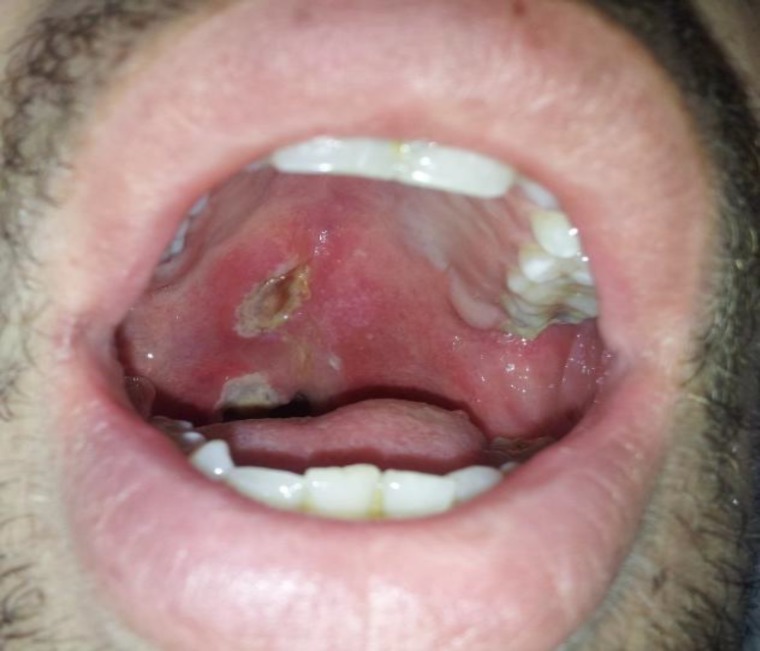
destruction of uvula with a destructive ulcer of the soft palate with a fibrinoleukocyter exudate.

In April 2015, a 23-year-old male was referred to our Faculty (Oral and Maxillofacial disease department of Mashhad university of Medical sciences). His chief complaint was an ulcer of the uvulae from three months ago, oropharyngeal dysphagia, pharyngitis, and mild fever with unintentional 20-pound weight loss over the course of 4 months. Also he was on a mechanical soft diet. Over the past four years, he had severe episodes of Pharyngitis without response to the usual administered drugs, as well as a history of severe dental infection involving the brain. He did not have any systemic disorders and no history of smoking, abusing drugs or drinking alcohol. He was a construction worker and often dealt with rock wool. Upon physical examination, two painful destructive ulcers were observed. One of them was about 2cm × 1 cm, extending from the soft palate to hard palate, exposing the underlying bone and another one was destructing the uvulae (Fig.1). The ulcers were covered with a thick fibrinoleukocyter membrane with erythematous borders. He had no history of such lesions. His oral hygiene was in good condition. Cervical lymph nodes were not appreciable. Disseminated hypopigmented maculopapular rash throughout the skin, from nine years ago, was also observed. Examination of vital signs revealed a blood pressure of 110/70 mm Hg, a heart rate of 80 beats per minute, and a body temperature of 100°F. The rest of his physical examination did not raise any concerns.

The patient had been initially admitted to the Ear, Nose and Throat department two months ago. Due to suspicion of an infective condition (Trench mouth), some antibiotics such as Ceftriaxone, Metronidazole and Clindamycin were administered for about one month but without significant improvement.

Because of the negative response to the drugs mentioned above the suspicion of an immunosuppressive condition was raised. Therefore, the patient was admitted to the allergy and immunology department. After consultation with an infectious disease specialist, the administered antibiotics were altered to Ceftazidime, Vancomycin, and Metronidazole. However, despite taking these remedies for about three weeks, no improvement was observed. Along with these treatments, some laboratory tests and paraclinical modalities had been requested below:

The blood test only showed Neutrophilia (about 80% of the white blood cell count) and Lymphocytopenia. Erythrocyte sedimentation rate was high and C-reactive protein was positive. Serum chemistry parameters were in normal range. All of the virology, microbiology and parasitology tests; such as Anti-HIV, Tuberculin skin testing, etc.; were negative. He had a high Lactate dehydrogenase level of about 595 IU/L. Given the patient's fevers, history of recurrent upper respiratory tract infection with depigmented skin rashes, Infectious mononucleosis was a concern when white blood cell morphology was studied and viral type Lympho-Mono were seen. Also viral capsid antigen (VCA)-IgM and VCA-IgG were dramatically high in the patient’s serum. The results of nitro blue Tetrazolium test NBT and Dihydrorhodamine Flow cytometric Assay (DHR) and other immunological laboratory findings were in normal ranges, except for high IgE (2375 µ/dl) and a slightly low IgM (25 mg/dl). Consequently Immunodeficiency and autoimmune disease were not confirmed. Paraclinical modalities such as Abdominal, Inguinal and Neck ultrasonography, Chest X-ray, Posterior-Anterior Skull X-ray were requested and no abnormalities were observed, except for multiple reactive lymph nodes up to 12 millimeters in size at the anterior cervical lymph node chain, which were detected by ultrasonography. Computed tomography (CT) scan of the head and neck with contrast medium revealed nothing of note. In addition, biopsy from palatal ulcers with fungal culture revealed a non-specific ulcer with extensive necrosis and bacterial colonies, fibrin deposition and vasculitis. Dysplasia, malignancy any fungal elements were not evident.

**Fig 2:**
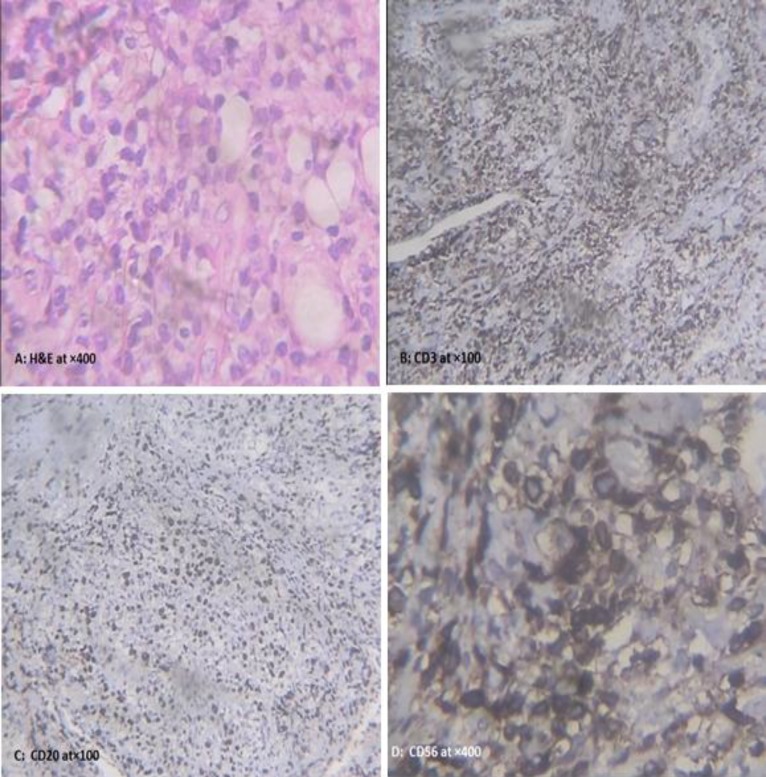
Hematoxylin and Eosin staining of the specimen at ×400 shows lymphoid cells with atypia, irregular and prominent nuclei in the background of the tissue. The benign inflammatory cell infiltrations with geographic necrosis in the background are evident. Immunohistochemical staining of the specimen (B toD) shows

A biopsy of the palatal ulcers was repeated and the histopathological examination of the specimen showed very dense lymphocyte infiltration. In the background of the tissue, lymphoid cell with atypia, irregular and prominent nuclei were seen. The benign inflammatory cell infiltration with geographic necrosis in the background was evident (Figure 2-A). After consultation, the patient was referred to our Faculty and two clinical provisional diagnoses were made: The first one was a Lymphoproliferative disease such as Non-Hodgkin lymphoma and the second one was necrotizing stomatitis accompagnied by immunocompromised conditions and viral infections like EBV, cytomegaloviruses (CMV) and Herpes simplex viruses (HSV). Hence immunohistochemical analysis of paraffin blocks was requested. CD3 was expressed in some background lymphoid cells, CD56 was positive in some lymphoid cells, CD20 was positive in some background lymphocytes, and the Ki-67 marker was positive in 60% of background of Lymphoid cells. Terminal deoxynucleotidyl transferase (TdT) was negative and finally the diagnosis of ENKL was confirmed (Figure 2-B to D). Clinical staging of the lymphoma according to the Ann Arbor classification was determined as stage IE (4). The patient was referred to the oncologist and a CHOP regimen of chemotherapy was administered every three weeks. After the third cycle of CHOP therapy, the patient unfortunately died due to sepsis and infection (about 3 months after treatment).

**2-B)** CD3 which was expressed in some background lymphoid cells

**2-C)** CD20 which is positive in some background lymphocytes and

**2-D)** CD56 which is positive in some lymphoid cells

## Discussion

NKTCL is a rare Non-Hodgkin's Lymphoma (NHL) which is more prevalent in Asia and South America and accounts for 7-10% of all non-Hodgkin lymphomas in these areas. Most cases have been reported from China, Taiwan, Korea, and Japan (5,6,9). Except for a case report of an Angiocentric Nasal T-Cell Lymphoma from Moradi et al. involving the palate, we couldn't find any other report of this condition in our country (Iran) (10).

One of the most common sites of extranodal NHL is Waldeyer ring, so the palate is a prevalent site for all the types of this malignancy (1,11). As a unique form of NHL, NKTCL usually has local destructive behavior rather than a proliferative or expansible nature in comparison with other types of Non-Hodgkin’ Lymphoma (9,12). Accordingly, the dominant signs of NKTCL are regional progressive extension to the form of ulcer at the maxillofacial or nasopharyngeal structures with midline propensity. So the location in the midline of these structures provides a clue for the diagnosis of lymphoma (1-6,8,9,13,14). About 42 cases of NKTCL have been previously published with palatal involvement (Table.1) (1,3,4,6,9,10,15-23), however destruction of the uvulae in this patient patient was an unusual manifestation of the disease that has not been previously noted in literature (15,24).

**Table 1:** Cases of NKTCL with palatal involvement

Case No.	Report’s Year	Authors	Number of cases	Description of lesions	Age/ gender
**1**	2015	Kidwai (15)	1	left soft palate with some mucosal sloughing	25/M
**2**	2014	Lee (6)	1	destructive ulcer exposing the underlying bone	39/F
**3**	2014	Gu (16)	3		
**4**	2014	Ramanathan	2		M
**5**	2013	Bi (17)	12		
**6**	2013	Hmidi (4)	1		54/M
**7**	2013	Li (25)			
**8**	2012	Tababi (3)	3	Hard palate ulceration, with oronasal fistulae in one case	
**9**	2012	Chauchet (18)	9	bilateral nasal obstruction with hard palate destructive ulcer	
**10**	2012	Nikolaos (21)	1	Deep destructive ulcer creating oronasal fistulae	40/M
**11**	2011	Bhaat (9)	1	Hard palate ulceration, with oronasal fistulae	21/M
**12**	2011	Macdonald (19)	1		61/F
**13**	2010	Meng(20)	1	palatal ulcer as the earliest clinical feature	
**14**	2009	Moradi(10)	1		13/M
**15**	2006	Patel (22)	1		40/M
**16**	2000	Tsang	3		
**Total numbers**			About 42 cases		

The symptoms of NKTCL are related to the sites involved and are non-specific. This may lead to delays in early detection of the disease and misdiagnoses with other conditions. Therefore, we have listed some conditions that manifest in the oral cavity with non-recurrent chronic destructive ulcers (especially for the palate), mimicking NKTCL (Table.2) (1,4,9,11,12,26-33). We couldn’t find any documented ranking in the literature and this ranking is based on the author’s opinion and may have a different order from area to area. Although, in this case, all of these differential diagnoses should not be considered. In our patient, at first presentation, the disease was misdiagnosed as Trench mouth. Differentiation of NKTCL from the diverse group of periodontal diseases is of clinical importance. Necrotizing ulcerative gingivitis (NUG), previously known as Trench mouth (during World War I in the battlefield trenches), has a distinctive clinical pattern. 

**Table 2:** differential diagnosis of the destructive ulcers of the oral cavity

Cause of palatal destructive ulcers in order of prevalence	Clinical presentations	Other diagnostic features
**1- Necrotizing ulcerative gingivitis or periodontitis (1,12).**	Usually occurs in immunocompromised host, sore mouth, malodor, metallic taste, periodontitis, ulcers extend from periodontium and marginal gingiva.	Fusobacterium, Treponema species etc. Histopathological evaluation is non-specific.
**2- Necrotizing Sialometaplasia (1,12)**	Presence of potential predisposing factors in history including traumatic injuries, dental injections, ill-fitting dentures, etc. Unilateral palatal ulceration	At Microscopic evaluations acinar necrosis in early lesions, followed by associated squamous metaplasia of the salivary ducts are characteristic and the overall lobular architecture of the involved glands is still preserved
**3- Deep fungal ulcers (Mucormycosis) (12)**	Is noted especially in insulin-dependent diabetics and immunocompromised patients, Symptoms related to cranial nerve involvement (e.g., facial paralysis), a primary pulmonary infection may be noted.	The histopathological specimen shows necrosis and non-septate hyphae, which are best demonstrated by a Periodic acid–schiff stain or the methenamine silver stain.
**4- Wegener’s Granulomatosis disease(1,12)**	Nasal, Renal and Pulmonary manifestations is evident	P-ANCA and C-ANCA are positive, microscopic finding of necrotizing and granulomatous vasculitis
**5- Squamous Cell Carcinoma (SCC) (1,12)**	Ulcer with rolled border or a fungating, papillary or verruciform surface around its margin	SCC is characterized histopathologically by invasive islands and cords of malignant squamous epithelial cells
**6-Malignant salivary gland Tumors(26)**	Necrosis within a smooth surfaced unilateral mass	
**7- Cocaine-induced injury** **(9,30,32)**	History of cocaine abuse	
**8- Lymphoma (NKTCL) (1,9)**	It is located at the midline. Previously known as midline lethal granuloma	Immunohistochemistry with predominant CD56 and cytoplasmic CD3 positive phenotype
**9- Tuberculosis (29,33)**	Occurs in immunodeficiency conditions, hemoptysis, chronic cough, fever, night sweeting and respiratory symptoms	Tuberculin skin testing (PPD) is positive, in chest X- ray marked by a formation of a cavity in the lungs
**10- Tertiary Syphilis (27)**		

The interdental gingiva is erythematous, hemorrhagic, and edematous. A characteristic hallmark of NUG is a punched-out feature of interdental papilla with craterlike necrosis that is covered with a grayish pseudomembrane layer (1). Necrotizing Stomatitis (NS), in many instances, begins as NUG, which may extend from the gingiva to the surrounding tissues such as the palate, buccal or lingual soft tissues which exposes the underlying bone. When destruction of facial soft tissue structures occurs, the term Noma (Cancrum Oris) is adopted. Both NUG and Necrotizing Stomatitis have the same predisposing factors such as malnutrition or dehydration, poor oral hygiene, an immunocompromised condition, malignancy, etc. (1). However, in our patient, prior to the formation of the ulcers, he was in good nutritional status and had good dentition without any periodontal pocket or calculus, which made NUG unlikely. Further laboratory findings had proved no immunodeficiency which further decreased the doubt of NUG, NS and some chronic ulcers (listed in Table 2 such as deep fungal ulcers and Tuberculosis), which arise in an immunocompromised status. 

Some types of systemic vasculitides such as Wegener’s Granulomatosis disease (Granulomatosis with polyangiitis or GAP) have few oral manifestations such as ulcers (34-36). The most common oral presentation of GAP is gingival hyperplasia with a granular, hemorrhagic and friable pattern (strawberry gingivitis). Although an ulcerative pattern can usually occur at the end stage of GAP, when renal involvement is evident (1). As mentioned in Table 2, the three main presentations of GAP are upper and lower respiratory tract and renal symptoms, in addition to a positive ANCA test. However, none of these presentations existed in this case.In NKTCL, paraclinical evaluations such as magnetic resonance imaging (MRI) and computed tomography (CT) modalities are suggestive of a mass or defects in the aerodigestive tract structures and are utilized for tumor staging (4). However, in our case, these modalities showed nothing. 

Laboratory data are not conclusive but some serological tests may be contributive such as antibodies to EBV viral capsid antigen (VCA) IgG, IgA, and early antigen (EA) IgG (37). 

The diagnosis of NKTCL is largely based on laboratory findings.Clinical,immunephenotypic, histopathological, immunohistochemical, molecular and genetic findings must be correlated as none of them is strong enough to be used alone for diagnosis (38). Immunohisto- chemistry is the cornerstone of the definitive diagnosis of NKTCL which is typically CD2 +CD56 + surface CD3, cytoplasmic CD (CD3 epsilon) and cytotoxic granule-associated protein (TIA-1 ,granzyme B and perforin ) positive (9). Expression of the ki-67 is high in NKTCL and above 60% of patients are positive with this index, similarly to our patient (5) (Figure 2). The pathogenesis of NKTCL is unknown but the constant association with EBV has been proved in different studies across the world (39-41). Similarly, in our case, high titers of viral EBV capsid antigen were indicative of the relationship between NKTCL and EBV infection.

Furthermore, an immunocompromised status can play a role in the pathogenesis of NKTCL. Some reports of lymphoma have been observed in transplant patients or patients with human immunodeficiency virus(HIV)infections(40,42).

Additionally, smoking and contamination of drinking water with nitrate were noted as risk factors of Non-Hodgkin’s Lymphoma in some of the literature (43). Our patient was exposed to rock wool for about four years but the carcinogenicity of this material has not been proven yet. If rock wool could have carcinogenic properties, we would expect it to lead to the development of respiratory cancers rather than hematological ones (44-46). Treatment of ENKL depending on the stage of disease, age and comorbidity, consists of radiotherapy or chemo-therapy, either alone or combined (11). For early stages of common aggressive non-Hodgkin lymphoma, three to four courses of a chemotherapy regimen that includes Anthracy- cline, such as CHOP, accompanied by irradiation is regarded as the standard therapy (47).

## Conclusion

Early diagnosis of NKTCL plays an important role in the improvement of the prognosis and survival rate of the disease. Non-specific manifestations of NKTCL can lead to misdiagnoses with other clinical conditions such as infective diseases, etc. Therefore, being aware of differential diagnoses of palatal ulcers can prevent the delays in diagnoses of NKTCL of the oral cavity.
